# Dr. PIAS: an integrative system for assessing the druggability of protein-protein interactions

**DOI:** 10.1186/1471-2105-12-50

**Published:** 2011-02-09

**Authors:** Nobuyoshi Sugaya, Toshio Furuya

**Affiliations:** 1 Drug Discovery Department, Research & Development Division, PharmaDesign, Inc., Hatchobori 2-19-8, Chuo-ku, Tokyo, Japan

## Abstract

**Background:**

The amount of data on protein-protein interactions (PPIs) available in public databases and in the literature has rapidly expanded in recent years. PPI data can provide useful information for researchers in pharmacology and medicine as well as those in interactome studies. There is urgent need for a novel methodology or software allowing the efficient utilization of PPI data in pharmacology and medicine.

**Results:**

To address this need, we have developed the 'Druggable Protein-protein Interaction Assessment System' (Dr. PIAS). Dr. PIAS has a meta-database that stores various types of information (tertiary structures, drugs/chemicals, and biological functions associated with PPIs) retrieved from public sources. By integrating this information, Dr. PIAS assesses whether a PPI is druggable as a target for small chemical ligands by using a supervised machine-learning method, support vector machine (SVM). Dr. PIAS holds not only known druggable PPIs but also all PPIs of human, mouse, rat, and human immunodeficiency virus (HIV) proteins identified to date.

**Conclusions:**

The design concept of Dr. PIAS is distinct from other published PPI databases in that it focuses on selecting the PPIs most likely to make good drug targets, rather than merely collecting PPI data.

## Background

The importance of PPIs as targets for drugs, especially small molecule drugs, has increased greatly in recent years [1-4]. Over 30 PPIs have been widely studied as targets for PPI-inhibiting small ligands. Currently, a huge amount of PPI data has been rapidly accumulated in public databases and in the literature. In addition, advances in high-throughput experimental technologies have lead to a large amount of various types of omics data, which have been deposited in many databases. These PPI data and omics data require methodologies for their application to pharmacological and medicinal studies. There is an urgent need to identify novel PPIs as drug targets from the PPI data accumulated, since only about 30 druggable PPIs have been well studied to date, whereas approximately 60,000 PPIs have been identified in human. We have recently proposed integrative approaches for discovering drug target PPIs by assessing the druggability of PPIs by the use of various types of omics data [5,6]. The application of our methods to human PPIs predicted many potentially druggable PPIs.

Several databases and web-based tools specializing in drug targets have been published. For example, TTD [7,8], a database of known therapeutic target proteins, stores information relevant to the targets, such as tertiary structures, disease associations, pathways, and pertinent literature. PDTD [9], a database for *in silico *drug target identification, stores diverse information on drug target proteins identified by the web-based tool Target Fishing Docking. SuperPred [10], a web-server for drug classification, uses a similarity score between drugs/chemicals to predict drug target proteins. These drug target databases and web-servers are very useful for researchers in *in silico *pharmacology and medicine. All of them, however, deal only with single proteins, rather than PPIs.

Recently, two databases (2P2I_DB _[11] and TIMBAL [12]) specializing in drug target PPIs and PPI-inhibiting chemicals have been published. 2P2I_DB _mainly focuses on protein/protein and protein/inhibitor interfaces in terms of various physicochemical parameters such as atom and residue properties, pocket volume, and accessible surface area [11]. TIMBAL is a database of small molecules that inhibit protein/protein complexes, and it stores many properties of the molecules such as molecular weight, LogP value, number of rings, number of rotatable bonds, and binding affinity [12]. 2P2I_DB _and TIMBAL can provide useful information to researchers developing PPI inhibitors. Both databases, however, contain only known drug target PPIs, so only a very small number of PPIs and PPI-inhibiting chemicals are stored. As a next step, in order to efficiently utilize the databases such as 2P2I_DB _and TIMBAL, it is needed to apply the information obtained from known drug target PPIs and their inhibitors to other PPIs not presently targeted by inhibitors.

Here we describe a novel database system, Dr. PIAS, which focuses on the druggability of PPIs. Dr. PIAS assesses the druggability of PPIs, currently not targeted by inhibitors, by utilizing the information obtained from known drug target PPIs. Dr. PIAS holds not only known drug target PPIs but also all PPIs identified to date for human, mouse, rat, and HIV proteins. In addition to information on the properties of the tertiary structures of PPI interfaces and that on the properties of drugs/chemicals related to interacting proteins, which are dealt with in 2P2I_DB _and TIMBAL, other properties associated with the biological function of PPIs are also included in the assessment. This is important because, to select a drug target PPI, a researcher considers not only information on the tertiary structure of the PPI and its known inhibitors but also that on the biological function of the PPI. All information on the PPIs used in the assessment is stored in Dr. PIAS. Users can search for druggable PPIs in Dr. PIAS by using various words and terms such as protein/gene name, tertiary structure, disease, pathway, and drug/chemical name as keywords.

## Construction and context

### Assessing the druggability of PPIs

The most distinctive characteristic of Dr. PIAS is that the system assesses the druggability of PPIs by our original SVM-based method [6]. Thirty known drug target PPIs, including IL2/IL2RA, MDM2/TP53, and BCL2/BAK1, serve as the positive instances (Additional file 1: Table S1). These PPIs were selected from review articles focusing on druggable PPIs [1-4,13-15]. Positive instances must satisfy both the following two criteria.

• A PPI-inhibiting small chemical has been identified, and its potency as a PPI inhibitor has been validated by *in vitro *and/or *in vivo *assays.

• A binding pocket for the PPI-inhibiting small chemical has been located on the tertiary structure of a protein, and it overlaps with the PPI interface.

Structural, drug/chemical, and functional attributes (Additional file 1: Table S2) of the positive instances and other PPIs in Dr. PIAS (test instances) were calculated and stored in Dr. PIAS. We used these attributes for our SVM-based method [6]. The program package Libsvm [16] was used for SVM.

In previous study, we have obtained the best SVM model for discriminating the positive and negative instances, when the radial basis function kernel and the ratio of positives:negatives = 1:1 were used in machine learning by SVM [6]. The cross validation test using the best model showed the accuracy of 80.5% (sensitivity, 81.6%; specificity, 79.4%) that was comparable to the values of accuracy in previous studies on drug target prediction [6]. Also in Dr. PIAS, we adopt this SVM model for the assessment of the druggability of PPIs.

**Figure 1:**
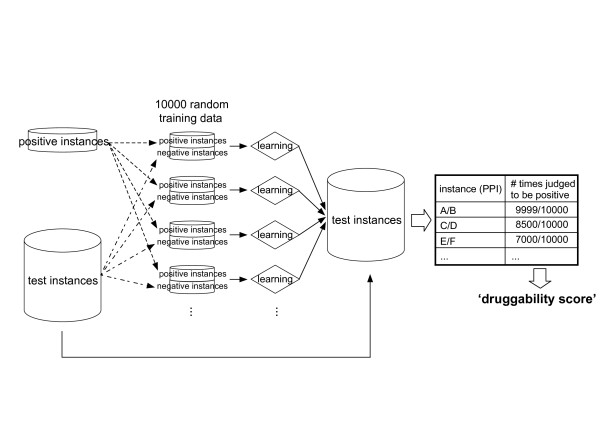
**Definition and calculation method of 'druggability score'**. The flow chart of calculating 'druggability score' by our SVM-based method is schematically illustrated. For details, see text.

We defined 'druggability score' to quantitatively assess the druggability of PPIs [6]. Druggability score is based on the results of our SVM-based method (Figure 1). To conduct machine learning by SVM, we created training data from the positive and negative instances. The ratio of positives to negatives was set as 1:1. The negative instances were randomly chosen from the test instances, since it was very difficult to define a group of PPIs as 'negative'. In this study, 'negative' PPI can be PPI for which there is no chemical that inhibit the PPI. We cannot be certain at present that a small chemical inhibiting the PPI will not be discovered in future studies. To avoid any bias in choosing the negatives from the test instances, we created 10,000 random training data sets. To predict novel druggable PPIs, the SVM models trained by each of the 10,000 random training data sets were applied to the test instances. We counted the number of times an instance (or a PPI) was judged to be positive in the 10,000 training-prediction iterations. This number was divided by 10,000 and then was defined as the druggability score. The scores range from 0 (non-druggable) to 1 (highly druggable). For example, the score of 0.9999 of a PPI indicates that the PPI is judged to be positive by the 9,999 SVM models of the 10,000 models and that the PPI is predicted as 'highly druggable' (Figure 1). Because the negative instances are randomly chosen from the test instances, it is highly probable that negatives of one training data are composed of PPIs similar to the positives, while those of another training data are composed of PPIs dissimilar to the positives. A high druggability score of a PPI means that the PPI is similar to the positives, no matter what type of PPIs constitutes the negatives. Thus, the higher the score of a PPI, the more likely the PPI has attributes similar to those of the positive instances (known drug target PPIs).

### Data sources of PPIs

The PPI data stored in Dr. PIAS were retrieved from public sources [17,18] and from several studies on the identification of human PPIs by high-throughput experimental assays [19-21] (Table 1). As of 2010/12, Dr. PIAS contains 71,500 PPIs. Most of these PPIs (63,010/71,510; 88%) are between human proteins (Table 2). The number of PPIs between mouse proteins (3,331) and those between human and HIV proteins (2,295) follows that of human PPIs. As shown below, Dr. PIAS has a meta-database that stores various types of information (tertiary structures, drugs/chemicals, and biological function associated with PPIs) retrieved from public sources (Table 3).

**Table 1:** Data source of PPIs stored in Dr. PIAS.

Data source	Number of PPIs	Reference
Entrez Gene	57,570	[17]
Ramani et al. (2008)	6,937	[21]
Genome Network Platform	5,528	[18]
Stelzl et al. (2005)	3,164	[19]
Lim et al. (2006)	2,296	[20]
		
Total number of non-redundant PPIs	71,500	

**Table 2:** Number of PPIs from each species stored in Dr. PIAS.

Species	Number of PPIs
Human	63,010
Mouse	3,331
HIV^a^	2,295
Rat	870
Others	1,994

^a^PPIs between human and HIV proteins.

**Table 3:** Information stored in Dr. PIAS.

Information	Source(s) or program(s) used
Structural information	
Tertiary structures	[22-24]
Pockets on PPI interface	[25]
Volume	[6,25]
Accessible surface area	[6,25]
Compactness	[6]
Planarity	[6]
Narrowness	[6]
Curvature	[26]
Roughness	[26]
Amino acid composition	[6,24,25,27]
Domains	[28,29]
Disordered regions	[37]
Amino acid sequence motifs	[38,39]
	
Drug/chemical information	
FDA-approved drugs	[40]
Chemicals associated with interacting proteins	[41]
	
Functional information	
Human diseases in OMIM	[42]
Number of interacting proteins in PPI network	[6]
Biological pathways	[43,44]
GO terms and identity scores of GO terms	[6,45]
Gene expression profiles and similarity scores of profiles	[6,46]
Paralogs	[43,47]

### Structural information

Several properties of PPIs stored in Dr. PIAS were pre-calculated using our original and several freely available computational algorithms/programs. Among the properties of PPIs, those based on tertiary structure are the most important for *in silico *drug design and development. We checked whether the tertiary structure of the protein/protein complex of a PPI had been already solved. Amino acid sequence similarity searches using the computational program BLASTP [22] were conducted against the PDB database [23]. If both the two interacting proteins showed sequence identities of ≥80% to distinct polypeptide chains in the same PDB entry, and the two chains physically contact each other in the tertiary structure of the protein/protein complex, the PDB entry was considered to be the tertiary structure of the PPI. Whether two chains physically contact was checked by consulting the PPI interface information in the PDBsum database [24].

If the tertiary structure of a PPI had been already solved, we further detected potential ligand-binding pockets that overlap with the PPI interface by using the alpha site finder implemented in the software package Molecular Operating Environment [25]. Physicochemical and shape properties of the pockets were calculated and stored in our database (Table 3). Planarity, narrowness, and roughness of the pockets were originally defined by us [6,26]. These properties and compactness, curvature, and amino acid composition of the pockets were calculated by using computational programs/algorithms written by us [6,26]. Other properties were calculated by using the Molecular Operating Environment or obtained from the results of the computational program DSSP [27].

We retrieved information on protein domains from the Pfam database [28] and detected domains responsible for PPIs by consulting the iPfam database [29]. Lists of the interacting domain pair(s) and domains of each interacting protein are stored in our database.

Recent studies have revealed that, in some groups of PPIs, disordered regions of proteins and amino acid sequence motifs in these regions are responsible for PPIs [30-33]. Among the PPIs used in the positive instances in our SVM-based method, interfaces of some PPIs such as BCL2/BAK1, BIRC4/CASP9, and MDM2/TP53 are formed by interaction between an ordered region in one protein and a disordered region in the other protein. Disordered regions in BAK1, CASP9, and TP53 in monomer changed to ordered state when protein/protein complex is formed [34]. Some chemicals inhibiting these PPIs mimic sequence motifs in the disordered regions [34-36]. We predicted disordered regions using the computational program POODLE-L [37] and retrieved the information on sequence motifs from the ELM database [38]. If the number of 'instance's of a motif in ELM was ≥2, we manually made a multiple alignment of the instances, and then created a hidden Markov model profile by using the computational program HMMER [39]. Using the hidden Markov model profiles, the motifs were predicted for each protein by the HMMER. Lists of the motifs and disordered regions predicted by the programs are stored in our database.

### Drug/chemical information

In several of the known target PPIs such as ESR1/NCOA2 and GRB2/EGFR, one interacting partner (ESR1 and EGFR) is a druggable protein that has been already targeted by a drug approved by the United States Food and Drug Administration (FDA). Several other known target PPIs are novel drug targets, and both interacting partners have no FDA-approved drug targeting them. In our SVM-based method, we used the number of drugs as the PPI attributes to assess whether the fact that an interacting protein has been already targeted by existing drugs influences the selection of PPIs as drug targets. The information on the FDA-approved drugs was retrieved from the DrugBank database [40]. The number of drugs targeting each interacting partner protein of a PPI was counted and stored in our database together with lists of drugs.

The information on chemicals that experimentally assayed for the activity to each of the two interacting proteins was retrieved from the ChEMBL database [41].

### Functional information

Information on human diseases caused when a protein is heritably or somatically mutated is essential for assessing the druggability of the protein. We retrieved information on human diseases from the OMIM database [42]. When using the information in our SVM-based method, it was transformed to a score of 0 or 1 [6]. The information is scored as 1 if both two interacting proteins of a PPI are implicated in OMIM-registered diseases (not limited to the same disease). The attribute is scored as 0 if only one interacting protein is implicated in a disease or if neither interacting protein is implicated in diseases. Lists of diseases associated with each interacting protein are stored in our database.

To repress a disease state with drugs, it would be desirable in some cases to target the proteins that function as 'hubs' in a PPI network. In other cases, targeting proteins that function in a peripheral part of a network could be more feasible for the treatment of a disease. After the PPI network was constructed based on the PPI data stored in Dr. PIAS, the number of all interacting proteins for each partner of a PPI was counted and stored in our database.

As with the number of interacting proteins, proteins that function in a large number of biological pathways may be more desirable targets for therapeutic intervention for some diseases, while proteins involved in a limited number of pathways may be more desirable targets for other diseases. We retrieved information on biological pathways from the KEGG [43] and PID [44] databases. We counted the number of pathways in which a protein is involved and stored these numbers in our database together with lists of pathways.

To assess the degree of similarity in biological function of the two interacting proteins, we utilized Gene Ontology (GO) [45]. GO terms assigned to proteins in Dr. PIAS were retrieved from the GO database [45]. Based on the GO terms, we calculated the identity scores of GO terms between the two interacting proteins according to equation S3 in Additional file four in [6]. The identity scores and lists of GO terms assigned to each protein are stored in our database.

For a protein to be selected as a drug target, it is advantageous if the protein functions in a limited number of tissues/organs including the tissues/organs that develop the disease. Because of a scarcity of information on protein expression profiles in human, we utilized gene expression profiles. Information on gene expression profiles was retrieved from the UniGene database [46]. To assess the degree of similarity between the gene expression profiles of the two interacting proteins in a PPI, we calculated the similarity scores of expression profiles between the two genes according to equation S4 in Additional file 4 in [6]. The similarity scores and gene expression profiles are stored in our database.

The number of paralogs is an important factor in order for a protein to be selected as a drug target, since the researchers must consider potential adverse effects caused by the drug binding to non-target paralogs. Target proteins with a large number of paralogs may be associated with more severe adverse effects. The information on paralogs was retrieved from the KEGG and PIRSF [47] databases. We stored the number of paralogs of each protein and lists of paralogs in our database.

All information described above is stored in a relational database (MySQL).

## Utility

### User interface

**Figure 2:**
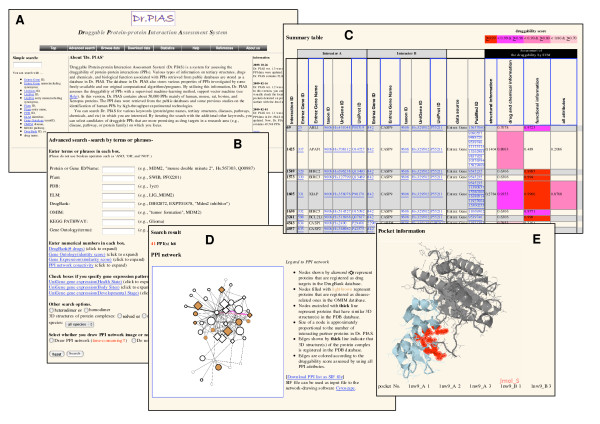
**Screenshots of Dr. PIAS**. (A) Top page of Dr. PIAS. (B) 'Advanced search' form. (C) Main search result summarizing PPIs and their druggability scores. (D) PPI network derived from a search result of Dr. PIAS. The network is drawn using the software eXpanda [48]. (E) Example of potential ligand-binding pocket on a PPI interface, which was used for assessing the druggability of a PPI. The pocket is shown by atoms colored red. The image is generated using the software Jmol [49]. (To see the pocket information, click the header 'structural information' in the main search result table, then click the header '# protein complexes in PDB' in the 'Structural information' table in a new window/tab opened, and then click '[pocket]' after the list of PDB entry names in the 'PDB information' table in a new window/tab opened.)

Screenshots of Dr. PIAS are shown in Figure 2. Figure 2A shows a top page from Dr. PIAS. Using the 'Advanced search' form of Dr. PIAS (Figure 2B), users can search for druggable PPIs in Dr. PIAS by inputting various keywords and terms (protein/gene names, tertiary structures, domains, motifs, diseases, pathways, GO terms, gene expression patterns, drug/chemical names, identifiers of public databases, *etc*). Users can also use the amino acid sequence of a protein as a query. Amino acid sequence similarity search is performed by the BLASTP program, and then PPIs of the query protein and those of homologs of the query are listed as a search result.

Figure 2C shows a main search result summarizing PPIs and their druggability scores. The columns of the druggability score are colored depending on the score (≥0.99, red; <0.99 and ≥0.9, magenta; <0.9 and ≥0.8, hotpink; <0.8 and ≥0.7, pink). The threshold of the coloring of ≥0.9 (red and magenta) is set based on the average value of the druggability scores of the positive instances (known drug target PPIs) used in the druggability assessment (see Figure 5 in [6]). Other thresholds are set arbitrarily. The assessment of the druggability of PPIs is conducted in four ways: three of the four use only the structural, drug/chemical, or functional attributes of the PPIs, respectively, while the fourth uses all attributes in our SVM-based method (Additional file 1: Table S2). These four ways correspond to the four columns of the druggability score in Figure 2C.

If a user selects the 'Draw PPI network' radio button in the 'Advanced search' form, a PPI network derived from the search result is drawn by the computational program eXpanda [48] (Figure 2D). Nodes and edges of the network represent proteins and PPIs, respectively. The PPI network reflects information stored in Dr. PIAS. If the tertiary structure of the protein/protein complex of a PPI has been solved, the edge representing the PPI is shown as a thick line. Edges of the network are colored according to the druggability score (calculated using all attributes). The size of the nodes is approximately proportional to the number of interacting partner proteins in Dr. PIAS. Nodes circled with a thick line represent proteins whose tertiary structures (but not necessarily those of the protein/protein complex) have been already solved. Nodes colored lightbrown represent proteins that are registered as disease-implicated in OMIM. Nodes shown by a diamond shape indicate proteins registered as known drug targets in DrugBank.

By clicking the headers of the summary table shown in Figure 2C, users can see details of the information stored in Dr. PIAS. Figure 2E is an example of the level of detail regarding a potential ligand-binding pocket on a PPI interface, which is used for assessing the druggability of a PPI. The pocket is shown by atoms colored red. The image was generated by using the software Jmol [49].

### Case studies

In this section, the usage of Dr. PIAS is illustrated by applying it to search for potentially druggable PPIs from two points of view: one is from a disease point of view and the other is from a protein.

#### Searching for druggable PPIs implicated in lung cancer

Lung cancer is one of the most common cancers in both men and women worldwide [50]. Many studies to develop drugs for the treatment of lung cancer have been conducted both in the pharmaceutical industry and in academia, but only a few drugs have been approved by the FDA [51,52]. The identification of novel drug targets for lung caner would prompt the development of drugs targeting this cancer.

**Figure 3:**
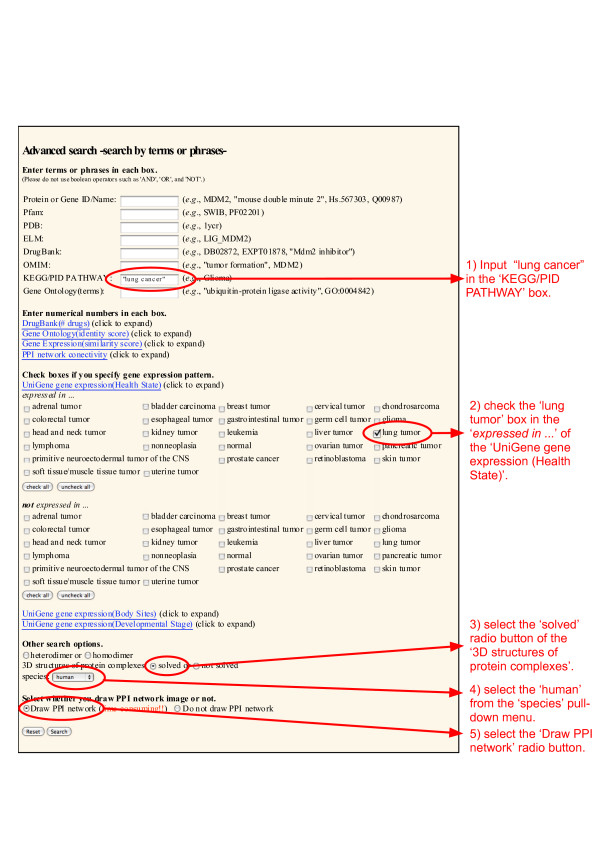
**Searching for PPIs implicated in lung cancer**. In the 'Advanced search' form of Dr. PIAS, 1) input ' "lung cancer" ' in the 'KEGG/PID PATHWAY' box, 2) check the 'lung tumor' box in the '*expressed in.*.' part of the 'UniGene gene expression (Health State)', 3) select the 'solved' radio button of the '3 D structures of protein complexes', 4) select the 'human' from the 'species' pull-down menu, and 5) select the 'Draw PPI network' radio button.

When using Dr. PIAS, users can easily set versatile criteria to search for druggable PPIs. To search for PPIs that are potentially druggable for lung cancer, we set the following criteria (Figure 3).

• A PPI is derived from human.

• The PPI is involved in biological pathways associated with lung caner.

• Genes coding interacting proteins are expressed in lung cancer cells.

• Tertiary structure of the protein complex of the PPI has been solved.

**Figure 4:**
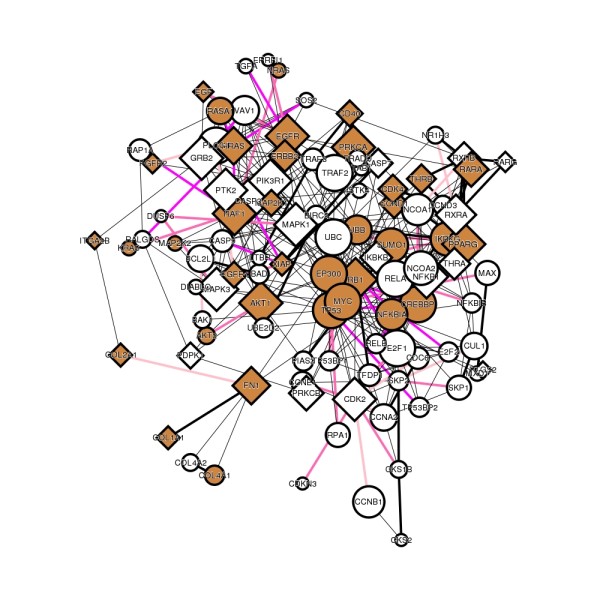
**PPI network related to lung cancer**. The network is drawn from the search result obtained from the criteria in Figure 3. Edges (PPIs) are colored according to their druggability scores (≥0.99, red; <0.99 and ≥0.9, magenta; <0.9 and ≥0.8, hotpink; <0.8 and ≥0.7, pink). For details of the coloring and shape of the nodes and edges, see text.

The last criterion is intended to search for PPIs that are more amenable to *in silico *drug design and development. If a user does not intend to design and develop drugs by *in silico *technologies, this criterion is superfluous. In total, 116 PPIs satisfy all the above criteria (Additional file 1: Table S3). The PPI network in Figure 4, drawn from the search result, shows that 17 PPIs in the network are assessed as highly druggable (druggability score ≥0.9, edges colored magenta) (Table 4).

**Table 4:** List of PPIs assessed as potentially druggable for lung cancer.

PPI	Druggability score			
	
	Structural attributes	Drug/chemical attributes	Functional attributes	All attributes
CREBBP/TP53	0.9747	0.2234	0.9682	0.9507
E2F1/RB1	0.6162	0.4677	0.9922	0.9402
E2F2/RB1	0.8452	0.4677	0.9964	0.9000
EGFR/TGFA	0.6832	0.7028	0.9957	0.9393
EP300/TP53	0.9747	0.2234	0.9214	0.9454
GRB2/GRB2	0.9327	0.2356	0.9838	0.9058
GRB2/VAV1	0.7431	0.2234	0.9978	0.9662
HRAS/RAF1	0.5453	0.3029	0.9973	0.9140
HRAS/RALGDS	0.9320	0.2234	0.9891	0.9152
HRAS/RASA1	0.7267	0.2234	0.9988	0.9459
HRAS/SOS2	0.8991	0.2234	0.9911	0.9293
NFKB1/NFKBIA	0.8607	0.8146	0.9993	0.9343
NFKB1/RELB	0.5218	0.8146	0.9868	0.9195
RAF1/RAP1A	0.8070	0.4380	0.9937	0.9154
TP53/TP53BP2	0.2102	0.2234	0.3776	0.9274
XIAP/CASP3	0.5897	0.3679	0.9998	0.9219
XIAP/CASP9	0.7910	0.2277	0.9954	0.9468

From the 116 PPIs searched in Dr. PIAS using the keywords and options, the PPIs having a druggability score of ≥0.9 in the 'All attributes' column are listed.

**Figure 5:**
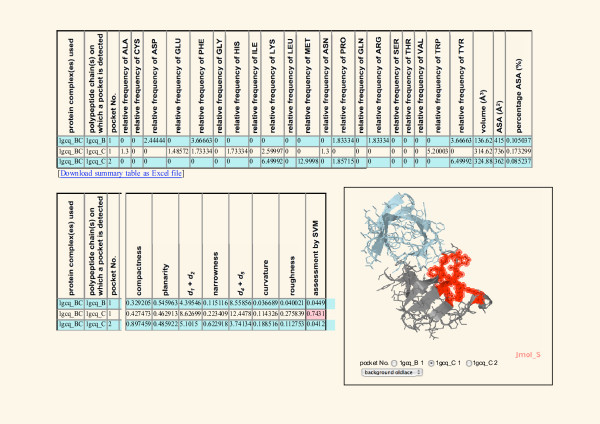
**Physicochemical and shape properties of the potential ligand-binding pockets located on GRB2/VAV1 PPI interface**. Three pockets were detected on the interface of GRB2/VAV1 complex [PDB:1GCQ]. The physicochemical and shape properties of the pockets are shown. These properties were used for the assessment of the druggability. For the definition and details of the properties, see [6,26]. The druggability scores calculated are shown in the column named 'assessment by SVM'. The pocket No. 1 identified on 1GCQ_C polypeptide chain has the highest score of 0.7431 (see Table 4). The location of this pocket is illustrated using the Jmol. In the illustration, the polypeptide chain 1GCQ_B is colored lightblue and 1GCQ_C is gray. The atoms constituting the pocket are colored red.

Among the 17 PPIs, GRB2/VAV1 has the highest score of 0.9662 when all attributes were used for the assessment. Figure 5 shows that 3 pockets were detected on GRB2/VAV1 interface and one of them (pocket No. 1 on 1GCQ_C polypeptide chain) has the highest score of 0.7141 when only structural attributes were used for the assessment. Compared with amino acid frequencies on the total surface of the protein, the pocket with the highest score are enriched in glutamic acid, phenylalanine, histidine, and tryptophan that are frequently observed as hot spots on PPI interfaces [53,54]. The accessible surface area (736Å^2^) of this pocket is the largest among the three pockets, and the volume (314.62Å^3^) is the second largest. GRB2 protein is a well-studied drug target [55]. One FDA-approved drug [DrugBank:DB00061] exists targeting GRB2, and 365 chemicals (as of 2010/12) experimentally assayed for the activities to GRB2 are registered in ChEMBL. In contrast, there is no approved drug and experimentally assayed chemical for VAV1 protein. Chemicals inhibiting GRB2/VAV1 PPI have not been reported, thus this PPI is a novel drug target.

Interestingly, the list of PPIs in Table 4 includes EGFR/TGFA, and the PPI has a high druggability score of 0.9393. TGFA is a member of the EGF family, and has a tertiary structure similar to EGF. TGFA forms a protein/protein complex with EGFR in a manner similar to EGFR/EGF complex [56]. EGFR/EGF PPI is the target for the FDA-approved anti-EGFR antibody, cetuximab, for the treatment of several cancers such as colorectal cancer and head and neck cancer [51,52]. EGFR/EGF is assessed as highly druggable by our method in three of the four assessments. A druggability score of 0.8712 is obtained when only structural attributes are used, whereas a score of 0.9997 is obtained when functional attributes are used, and a score of 0.8724 when all attributes are used (Additional file 1: Table S3). Thus, EGFR/TGFA may be a drug target for cancers including lung cancer. In Table S3, another PPI, ERBB2(HER2)/ERBB2(HER2), which has been already targeted by a FDA-approved drug, is included. The anti-HER2 antibody, trastuzumab, has been developed for the therapeutic intervention of breast cancer [51,52]. Although these FDA-approved drugs, cetuximab and trastuzumab, are not small molecule drugs, these results indicate that Dr. PIAS is effective in predicting druggable PPIs. PPIs in Table 4 other than GRB2/VAV1 and EGFR/TGFA may be potential drug targets for lung cancer in future studies.

### Searching for druggable PPIs associated with BCL-X_L_

As described in the former section, 2P2I_DB _and TIMBAL have been already published that hold drug target PPIs and their inhibitors. By using an identical protein as a query when searching for druggable PPIs in Dr. PIAS and the two databases, we demonstrate the differences and similarities in output results obtained from the three databases.

BCL-X_L _protein was adopted as a query. The PPIs of BCL-X_L _with BAX, BAK, and BID proteins are well-studied drug target PPIs [35], and both 2P2I_DB _and TIMBAL contain the PPIs. From 2P2I_DB_, a user can obtain the information on 8 tertiary structures (as of 2010/12) of BCL-X_L_/BAK protein/protein and BCL-X_L_/inhibitor complexes and the information on various physicochemical properties of the inhibitor-binding pockets. If a user search for BCL-X_L _in TIMBAL, the user can obtain the information on 26 chemicals (as of 2010/12) inhibiting the PPIs of BCL-X_L _with BAX, BAK, and BID. The two databases provide users with the structural and drug/chemical information on already-studied drug target PPIs.

When a user use the protein name 'BCL2L1' (a synonym of BCL-X_L_) as a keyword in searching Dr. PIAS, the user can obtain 66 PPIs (as of 2010/12) of BCL-X_L _with many other proteins as well as with BAX, BAK, and BID (Additional file 1: Table S4). Distinct from 2P2I_DB _and TIMBAL, Dr. PIAS assesses the druggability of all PPIs associated with BCL-X_L _as well as already-studied drug target PPIs and provides structural, drug/chemical, and functional information on all PPIs. Among the 66 PPIs, 5 PPIs (BAD/BCL2L1, BAK1/BCL2L1, BCL2L1/BCL2L1, BCL2L1/BCL2L11, and BCL2L1/Bcl2l11) have the tertiary structures of protein/protein complexes in the PDB, and the druggability assessments were conducted in all four ways (Additional file 1: Table S4). BAK1/BCL2L1 has the highest druggability score of 0.7674 when all attributes were used for the assessment. Like 2P2I_DB_, the information on the physicochemical and shape properties of the ligand-binding pocket on BAK1/BCL2L1 PPI interface can be obtained from Dr. PIAS (Table 3). Like TIMBAL, a user can obtain the drug/chemical information from Dr. PIAS. Unlike 2P2I_DB _and TIMBAL, Dr. PIAS provide a user with the information on biological function of BAK1 and BCL2L1 proteins (Table 3). BCL2L1 is involved in apoptosis pathway, focal adhesion pathway, and some cancer-related pathways (based on KEGG). BAK1 functions as a direct p53 effector (based on PID). The gene encoding BCL2L1 is expressed in many body sites (34/44 in UniGene). The gene encoding BAK1 is also the case (32/44 in UniGene). In PIRSF, BCL2L1 has 10 paralogs, while BAK1 has 5 paralogs. In addition to the structural and drug/chemical information, the functional information described above can be also helpful for a researcher to select drug target PPIs.

## Discussion

### Comparisons with existing databases

Currently, there exist two databases (2P2I_DB _and TIMBAL) that focus on drug target PPIs. These databases hold known drug target PPIs and their inhibitors. In contrast, Dr. PIAS holds all PPIs of human, mouse, rat, and HIV proteins identified to date as well as known drug target PPIs. For each PPI, we assessed the druggability by the SVM-based method by using structural, drug/chemical, and functional attributes of the PPIs. These two characteristics of Dr. PIAS are what make Dr. PIAS distinct from other existing databases. 2P2I_DB _and TIMBAL primarily focus on the structural aspects of protein pockets and the chemical properties of PPI-inhibiting ligands, respectively. The information on the biological function of a protein is also essential for selecting drug target PPIs. Dr. PIAS stores functional information on interacting proteins, such as disease associations, pathways, GO terms, gene expression profiles, and paralogs. Therefore, Dr. PIAS can help researchers select drug target PPIs by evaluating each PPI from the three viewpoints of the tertiary structures of protein/protein complexes, drugs/chemicals relevant to interacting proteins, and the biological roles of PPIs in living cells. A cross reference of the three databases can provide researchers with a synergistic power to prompt studies on drug target PPIs and chemicals inhibiting them.

Many databases of PPIs have been published, all with the purpose of collecting as many PPIs as possible from the literature and already-published similar databases. The main purpose for developing Dr. PIAS was not to merely collect PPI data but to select useful PPIs (in this case, as potential drug targets) from the collected data. In this sense, the design concept of Dr. PIAS is completely different from that of all other PPI databases published to date.

### Future development

The number of PPIs for which small molecule inhibitors have been discovered has gradually increased over the last decade. This trend will continue. As more information on known drug target PPIs accumulates, our assessment system based on information obtained from known target PPIs will be improved. We will intensively incorporate information on novel drug target PPIs to make Dr. PIAS more useful for researchers focusing on the development of PPI-inhibiting drugs. In addition, PPI data and all omics information will be updated every half year. The number of PPIs stored in Dr. PIAS will rapidly increase due to the accumulation of PPI data in public databases and in the literature.

## Conclusions

Dr. PIAS is a database system aimed at assessing the druggability of PPIs. Of the huge number of currently unidentified PPIs, there could be many latent PPIs that are highly druggable. Dr. PIAS will aid the efficient discovery of these druggable PPIs from the continuously growing amount of PPI data.

## Availability and requirements

Dr. PIAS is available at http://asp.gridasp.net/drpias/index.php. Academic non-profit users can freely access all of the contents stored in Dr. PIAS without paying a licensing fee. Commercial and for-profit users must obtain a license to access Dr. PIAS by paying a licensing fee to and entering into a license agreement with Beyond Computing, Co. Ltd. and PharmaDesign, Inc.

## List of abbreviations

Dr. PIAS: druggable protein-protein interaction assessment system; FDA: United States Food and Drug Administration; GO: gene ontology; HIV: human immunodeficiency virus; PPI: protein-protein interaction; SVM: support vector machine.

## Authors' contributions

NS conceived this study, developed the database and user-interface of Dr. PIAS, and drafted the manuscript. FT helped to draft the manuscript. All authors have read and approved the final manuscript.

## Supplementary Material

Additional file 1 **Supplementary tables**. Table S1 lists the positive set PPIs used in our SVM-based method to assess the druggability of PPIs. Table S2 is the list of the attributes of PPIs used in the assessment. Table S3 lists the PPIs satisfying the criteria set to search for potential drug targets for lung cancer (see text). Table S4 lists the PPIs associated with BCL-X**_L _**protein in Dr. PIAS. Click here for file
