# Supplementary material for: Dr. PIAS: an integrative system for assessing the druggability of protein-protein interactions
Source: BMC Bioinformatics. 2011 Feb 9;12:50. doi: 10.1186/1471-2105-12-50 (PMC3228542; doi:10.1186/1471-2105-12-50)
Supplement: Additional file 1 — Supplementary tables. Table S1 lists the positive set PPIs used in our SVM-based method to assess the druggability of PPIs. Table S2 is the list of the attributes of PPIs used in the assessment. Table S3 lists the PPIs satisfying the criteria set to search for potential drug targets for lung cancer (see text). Table S4 lists the PPIs associated with BCL-XL protein in Dr. PIAS. [file 1471-2105-12-50-S1.PDF]

Table S1: Positive set PPIs for the assessment of the druggability of PPIs.

PPI	PPI
ARF1/CYTH1	GRB2/MET
ARF1/CYTH2	HOXB1/PBX1
BCL2/BAK1	IL1B/IL1R1
BCL2L1/BAK1	IL2/IL2RA
BIRC4/CASP3	MAGI3/PTEN
BIRC4/CASP9	MDM2/TP53
BIRC4/DIABLO	PIK3R1/PDGFRB
BIRC5/BIRC5	RAC1/TIAM1
CALM1/CAMK1	RAC1/TRIO
CALM1/MYLK	STAT3/STAT3
CALM1/PDE1A	TCF7L1/CTNNB1
CD4/HLA-DQB1	TCF7L2/CTNNB1
ESR1/NCOA2	THRB/NCOA2
FKBP1A/TGFBR1	TNF/TNF
GRB2/EGFR	ZAP70/CD247

Table S2: Attributes of the PPIs used in the assessment of the druggability. [a]

Attribute
Structural attributes
Pocket volume
Accessible surface area of pocket
Percentage of accessible surface area of pocket to that of total surface of protein
Pocket compactness
Pocket planarity
d_1+d_2
Pocket narrowness
d_4+d_5
Pocket curvature
Pocket roughness
Ratio of Ala frequency on pocket surface to that on total surface [b]
Ratio of Cys frequency on pocket surface to that on total surface [b]
Ratio of Asp frequency on pocket surface to that on total surface [b]
Ratio of Glu frequency on pocket surface to that on total surface [b]
Ratio of Phe frequency on pocket surface to that on total surface [b]
Ratio of Gly frequency on pocket surface to that on total surface [b]
Ratio of His frequency on pocket surface to that on total surface [b]
Ratio of Ile frequency on pocket surface to that on total surface [b]
Ratio of Lys frequency on pocket surface to that on total surface [b]
Ratio of Leu frequency on pocket surface to that on total surface [b]
Ratio of Met frequency on pocket surface to that on total surface [b]
Ratio of Asn frequency on pocket surface to that on total surface [b]
Ratio of Pro frequency on pocket surface to that on total surface [b]
Ratio of Gln frequency on pocket surface to that on total surface [b]
Ratio of Arg frequency on pocket surface to that on total surface [b]
Ratio of Ser frequency on pocket surface to that on total surface [b]
Ratio of Thr frequency on pocket surface to that on total surface [b]
Ratio of Val frequency on pocket surface to that on total surface [b]
Ratio of Trp frequency on pocket surface to that on total surface [b]
Ratio of Tyr frequency on pocket surface to that on total surface [b]

Table S2: (continued)

Attribute
Drug/chemical attributes
Number of small chemical drugs in the DrugBank (*L*) [c]
Number of small chemical drugs in the DrugBank (*S*) [d]
Number of biotech drugs in the DrugBank (*L*) [c]
Number of biotech drugs in the DrugBank (*S*) [d]
Number of approved drugs in the DrugBank (*L*) [c]
Number of approved drugs in the DrugBank (*S*) [d]
Number of experimental drugs in the DrugBank (*L*) [c]
Number of experimental drugs in the DrugBank (*S*) [d]
Number of investigational drugs in the DrugBank (*L*) [c]
Number of investigational drugs in the DrugBank (*S*) [d]
Number of nutraceutical drugs in the DrugBank (*L*) [c]
Number of nutraceutical drugs in the DrugBank (*S*) [d]
Number of withdrawn drugs in the DrugBank (*L*) [c]
Number of withdrawn drugs in the DrugBank (*S*) [d]
Number of illicit drugs in the DrugBank (*L*) [c]
Number of illicit drugs in the DrugBank (*S*) [d]
Functional attributes
Both proteins are related to OMIM-registered diseases (1) or not (0)
Number of interacting proteins (*L*) [c]
Number of interacting proteins (*S*) [d]
Number of KEGG pathways in which either protein is involved (*L*) [c]
Number of KEGG pathways in which either protein is involved (*S*) [d]
Number of KEGG pathways in which both interacting proteins are involved
Number of PID pathways in which either protein is involved (*L*) [c]
Number of PID pathways in which either protein is involved (*S*) [d]
Number of PID pathways in which both interacting proteins are involved
Identity scores of the GO terms in the Cellular Component category
Identity scores of the GO terms in the Molecular Function category
Identity scores of the GO terms in the Biological Process category
Number of paralogs in the KEGG (*L*) [c]
Number of paralogs in the KEGG (*S*) [d]

Table S2: (continued)

Attribute
Functional attributes
Number of paralogs in the PIRSF (*L*) [c]
Number of paralogs in the PIRSF (*S*) [d]
Number of gene-expressing health states (*L*) [c]
Number of gene-expressing health states (*S*) [d]
Number of health states in which both genes are expressed
Number of gene-expressing body sites (*L*) [c]
Number of gene-expressing body sites (*S*) [d]
Number of body sites in which both genes are expressed
Number of gene-expressing developmental stages (*L*) [c]
Number of gene-expressing developmental stages (*S*) [d]
Number of developmental stages in which both genes are expressed
Similarity scores of gene expression profiles in the Health State category
Similarity scores of gene expression profiles in the Body Sites category
Similarity scores of gene expression profiles in the Developmental Stage category

[a]For details of the definitions and calculation methods, see Additional file 4 in [6] and help page of Dr. PIAS [26].

[b]Abbreviations: Ala, alanine; Cys, cysteine; Asp, aspartic acid; Glu, glutamic acid; Phe, phenylalanine; Gly, glycine; His, histidine; Ile, isoleucine; Lys, lysine; Leu, leucine; Met, methionine; Asn, asparagine; Pro, proline; Gln, glutamine; Arg, arginine; Ser, serine; Thr, threonine; Val, valine; Trp, tryptophan; Tyr, tyrosine.

[c]Defined as the larger one of the two numbers for the two interacting proteins in a PPI.

[d]Defined as the smaller one of the two numbers for the two interacting proteins in a PPI.

Table S3: List of PPIs satisfying the criteria set to search for potential drug targets for lung cancer.

PPI	Druggability score			
	Structural attributes	Drug/chemical attributes	Functional attributes	All attributes
AKT1/AKT1	0.6639	0.5624	0.9669	0.8021
AKT1/AKT2	0.6639	0.4075	0.9945	0.8433
AKT2/AKT2	0.6639	0.2234	0.9900	0.6849
BAD/BCL2L1	0.7551	0.2234	0.9934	0.5230
BAK1/BCL2L1	0.9317	0.2234	0.9834	0.7674
BCL2L1/BCL2L1	0.6102	0.2234	0.9990	0.3927
BIRC3/BIRC3	0.6542	0.2234	0.9954	0.5717
BIRC3/UBE2D2	0.2248	0.2234	0.7824	0.5169
CASP9/CASP9	0.8629	0.2234	0.9921	0.7050
CCNA2/CDK2	0.9290	0.6521	0.9336	0.7355
CCNB1/CDK2	0.6660	0.6521	0.9283	0.7151
CCND1/CDK4	0.9566	0.4666	0.9977	0.7980
CCND3/CDK4	0.8161	0.3405	0.9685	0.6466
CCNE1/CDK2	0.8111	0.6521	0.9988	0.8354
CD40/TRAF3	0.4988	0.2099	0.3657	0.5627
CDC6/CDK2	0.9152	0.6521	0.8065	0.7722
CDK2/CDK2	0.1659	0.6347	0.9959	0.7345
CDK2/CDKN3	0.6031	0.5239	0.4654	0.8400
CDK2/CKS1B	0.5161	0.5239	0.4741	0.8502
CKS1B/CKS1B	0.9353	0.2234	0.1834	0.2543
CKS1B/CKS2	0.9353	0.2234	0.0064	0.2358
CKS1B/SKP2	0.8796	0.2234	0.0289	0.2797
COL1A1/FN1	0.2889	0.5691	0.2404	0.6750
COL2A1/FN1	0.2889	0.6110	0.1583	0.7541
COL4A1/COL4A1	0.9643	0.2234	0.3538	0.2046
COL4A1/COL4A2	0.9622	0.2234	0.4991	0.1781
COL4A2/COL4A2	0.9148	0.2234	0.6128	0.3138
CREBBP/TP53	0.9747	0.2234	0.9682	0.9507
DUSP6/MAPK1	0.8519	0.4256	0.9851	0.8989
DUSP6/MAPK3	0.8519	0.4983	0.9871	0.8937

Table S3: (continued)

PPI	Druggability score			
	Structural attributes	Drug/chemical attributes	Functional attributes	All attributes
E2F1/E2F1	- [a]	0.2234	0.9980	- [a]
E2F1/RB1	0.6162	0.4677	0.9922	0.9402
E2F1/TFDP1	0.5469	0.2234	0.9929	0.8894
E2F2/RB1	0.8452	0.4677	0.9964	0.9000
EGF/EGF	- [a]	0.2356	1.0000	- [a]
EGF/EGFR	0.8712	0.5131	0.9997	0.8724
EGFR/EGFR	0.9638	0.6403	0.9979	0.7877
EGFR/TGFA	0.6832	0.7028	0.9957	0.9393
EGFR/ERRFI1	0.5423	0.7028	0.3513	0.8764
EP300/TP53	0.9747	0.2234	0.9214	0.9454
ERBB2/ERBB2	- [a]	0.5465	0.9962	- [a]
FGFR1/PLCG1	0.4560	0.2234	0.9827	0.7252
FGFR2/PLCG1	0.4560	0.2398	0.9228	0.7482
GRB2/GRB2	0.9327	0.2356	0.9838	0.9058
GRB2/VAV1	0.7431	0.2234	0.9978	0.9662
HRAS/RAF1	0.5453	0.3029	0.9973	0.9140
HRAS/RALGDS	0.9320	0.2234	0.9891	0.9152
HRAS/RASA1	0.7267	0.2234	0.9988	0.9459
HRAS/SOS2	0.8991	0.2234	0.9911	0.9293
IKBKB/IKBKB	- [a]	0.4348	0.9995	- [a]
IKBKB/IKBKG	0.0789	0.3919	0.9993	0.8370
IKBKG/IKBKG	0.3282	0.2234	0.9983	0.7282
ITGA2B/ITGA2B	0.8755	0.2356	0.9836	0.1345
KRAS/RAF1	0.5453	0.3029	0.9960	0.8904
KRAS/RALGDS	0.9320	0.2234	0.9314	0.8188
LTBR/TRAF3	0.4894	0.2234	0.2247	0.5815
MAP2K1/MAP2K1	0.6874	0.2356	0.9874	0.7581
MAP2K2/MAP2K2	0.6874	0.2234	0.9915	0.7174
MAPK1/MAPK1	0.8755	0.6429	0.9402	0.7443
MAPK3/MAPK3	0.8755	0.4987	0.9409	0.7624
MAX/MAX	0.5771	0.2234	0.9906	0.1573

Table S3: (continued)

PPI	Druggability score			
	Structural attributes	Drug/chemical attributes	Functional attributes	All attributes
MAX/MYC	0.5971	0.2234	0.9788	0.8929
MXD1/MAX	0.5295	0.2234	0.8969	0.5232
NFKB1/NFKB1	0.5485	0.7570	0.9972	0.8524
NFKB1/NFKBIA	0.8607	0.8146	0.9993	0.9343
NFKB1/RELA	0.8788	0.8146	0.9990	0.8905
NFKB1/RELB	0.5218	0.8146	0.9868	0.9195
NFKBIA/RELA	0.6738	0.2234	0.9979	0.8359
NFKBIB/RELA	0.6528	0.2234	0.9689	0.8386
NRAS/RAF1	0.5453	0.3029	0.9961	0.8868
PDPK1/PDPK1	0.4570	0.5019	0.9991	0.8241
PIK3R1/PIK3R1	0.7117	0.2356	0.9355	0.8365
PPARG/RXRA	0.9608	0.8830	0.8803	0.6803
PPARG/RXRB	0.9608	0.7257	0.5320	0.6262
PRKCA/PRKCA	0.8007	0.5757	0.9977	0.8491
PRKCB/PRKCB	0.8007	0.4325	0.9941	0.6469
PTGS2/PTGS2	0.8526	0.6518	0.9964	0.6337
PTK2/PTK2	0.8122	0.2356	0.9997	0.8511
RAF1/RAF1	0.8890	0.4325	0.9945	0.8845
RAF1/RAP1A	0.8070	0.4380	0.9937	0.9154
RARA/RXRA	0.9898	0.8281	0.6165	0.5643
RARA/RXRB	0.9898	0.7162	0.1066	0.3823
RARG/RXRA	0.9898	0.9070	0.7314	0.4611
RARG/RXRB	0.9898	0.7392	0.1789	0.3648
RB1/RB1	0.9183	0.3689	0.9883	0.7744
RB1/TFDP1	- [a]	0.2807	0.9847	- [a]
RELA/RELA	0.7043	0.2234	0.9988	0.8126
RPA1/TP53	0.0767	0.2234	0.6630	0.8530
RXRA/THRA	0.6715	0.8063	0.3939	0.4821
RXRA/THRB	0.6715	0.8114	0.6000	0.5134
RXRA/NCOA1	0.6127	0.6987	0.5560	0.8178
RXRA/NR1H3	0.9788	0.6987	0.9232	0.7167

Table S3: (continued)

PPI	Druggability score			
	Structural attributes	Drug/chemical attributes	Functional attributes	All attributes
RXRA/NCOA2	0.6580	0.6987	0.7559	0.6016
RXRA/RXRA	0.9960	0.8677	0.9793	0.5511
RXRB/RXRB	0.9960	0.7574	0.5794	0.4130
RXRB/THRA	0.6715	0.9123	0.0728	0.4101
RXRB/THRB	0.6715	0.8582	0.5789	0.5280
RXRB/NCOA1	0.6127	0.8823	0.0497	0.7327
RXRB/NR1H3	0.9788	0.8823	0.4556	0.5451
SKP1/SKP2	0.9619	0.2234	0.0019	0.8067
SKP2/CUL1	0.2924	0.2234	0.0116	0.7131
STK4/STK4	0.6960	0.2234	0.5546	0.1169
SUMO1/PIAS2	0.6592	0.2234	0.7617	0.2411
TP53/TP53	0.8170	0.2234	0.9635	0.8437
TP53/TP53BP1	0.5251	0.2234	0.4087	0.8858
TP53/TP53BP2	0.2102	0.2234	0.3776	0.9274
TRAF2/TRAF2	0.9009	0.2234	0.9549	0.7274
TRAF2/TRADD	0.6150	0.2234	0.9964	0.7887
UBB/IKBKG	0.8772	0.2234	0.3383	0.6541
UBC/IKBKG	0.8772	0.2234	0.2454	0.8513
XIAP/XIAP	0.9032	0.2881	0.9578	0.5464
XIAP/CASP3	0.5897	0.3679	0.9998	0.9219
XIAP/CASP7	0.8914	0.2881	0.9010	0.4266
XIAP/CASP9	0.7910	0.2277	0.9954	0.9468
XIAP/TAB1	0.5163	0.2277	0.9860	0.6750
XIAP/DIABLO	0.8875	0.2277	0.9449	0.8995

Druggability score calculated by the SVM-based method using structural, drug/chemical, functional, or all attributes are shown.

[a] '-' means that the druggability score can not be calculated because any pocket overlapped with PPI interface was not detected, thus structural attributes of pocket can not be obtained. In these cases, the druggability score using all attributes is also not calculated.

Table S4: List of PPIs associated with BCL-X_L protein in Dr. PIAS.

PPI	Druggability score			
	Structural attributes	Drug/chemical attributes	Functional attributes	All attributes
APAF1/BCL2L1	- [a]	0.4091	0.2618	- [a]
BAD/BCL2L1	0.7551	0.2234	0.9934	0.5230
BAG1/BCL2L1	- [a]	0.2234	0.0036	- [a]
BAK1/BCL2L1	0.9317	0.2234	0.9834	0.7674
BAX/BCL2L1	- [a]	0.2234	0.9884	- [a]
BCL2/BCL2L1	- [a]	0.5071	0.9999	- [a]
BCL2L1/BCL2L1	0.6102	0.2234	0.9990	0.3927
BCL2L1/BID	- [a]	0.2234	0.9623	- [a]
BCL2L1/BIK	- [a]	0.2234	0.1431	- [a]
BCL2L1/BLK	- [a]	0.2234	0.2702	- [a]
BCL2L1/BNIP1	- [a]	0.2234	0.6441	- [a]
BCL2L1/BNIP3	- [a]	0.2234	0.9842	- [a]
BCL2L1/BNIP3L	- [a]	0.2234	0.8174	- [a]
BCL2L1/CAPN1	- [a]	0.2796	0.3341	- [a]
BCL2L1/CASP1	- [a]	0.3405	0.8520	- [a]
BCL2L1/CASP2	- [a]	0.2234	0.5204	- [a]
BCL2L1/CASP8	- [a]	0.2234	0.9824	- [a]
BCL2L1/CASP9	- [a]	0.2234	0.9448	- [a]
BCL2L1/CRYAA	- [a]	0.2234	0.0431	- [a]
BCL2L1/CRYAB	- [a]	0.2234	0.3590	- [a]
BCL2L1/IRS1	- [a]	0.2234	0.2450	- [a]
BCL2L1/MCL1	- [a]	0.2234	0.9361	- [a]
BCL2L1/PMAIP1	- [a]	0.2234	0.0172	- [a]
BCL2L1/PPP1CA	- [a]	0.2234	0.7764	- [a]
BCL2L1/MAPK8	- [a]	0.2234	0.9908	- [a]
BCL2L1/PSEN1	- [a]	0.2234	0.9046	- [a]
BCL2L1/PSEN2	- [a]	0.2234	0.6291	- [a]
BCL2L1/RAD9A	- [a]	0.2234	0.0505	- [a]
BCL2L1/RAF1	- [a]	0.3029	0.9775	- [a]
BCL2L1/RTN1	- [a]	0.2234	0.0658	- [a]
BCL2L1/SNCA	- [a]	0.2796	0.2184	- [a]

Table S4: (continued)

PPI	Druggability score			
	Structural attributes	Drug/chemical attributes	Functional attributes	All attributes
BCL2L1/TMBIM6	-[a]	0.2234	0.2204	-[a]
BCL2L1/TP53	-[a]	0.2234	0.8387	-[a]
BCL2L1/TPT1	-[a]	0.2234	0.2592	-[a]
BCL2L1/VDAC1	-[a]	0.2796	0.8355	-[a]
BCL2L1/DYNLL1	-[a]	0.2234	0.0382	-[a]
BCL2L1/IRS2	-[a]	0.2234	0.0976	-[a]
BCL2L1/BECN1	-[a]	0.2234	0.0404	-[a]
BCL2L1/HRK	-[a]	0.2234	0.0380	-[a]
BCL2L1/CFLAR	-[a]	0.2234	0.3349	-[a]
BCL2L1/BCLAF1	-[a]	0.2234	0.0580	-[a]
BCL2L1/BCL2L10	-[a]	0.2234	0.2766	-[a]
BCL2L1/BCL2L11	0.7884	0.2234	0.8851	0.7218
BCL2L1/BCAP31	-[a]	0.2234	0.0328	-[a]
BCL2L1/SIVA1	-[a]	0.2234	0.0197	-[a]
BCL2L1/Bcl2l11	0.7707	0.2234	0.0000	0.0546
BCL2L1/IKZF3	-[a]	0.2234	0.0650	-[a]
BCL2L1/NLRP1	-[a]	0.2234	0.4746	-[a]
BCL2L1/FKBP8	-[a]	0.2234	0.4797	-[a]
BCL2L1/BBC3	-[a]	0.2234	0.7235	-[a]
BCL2L1/CYCS	-[a]	0.3405	0.8899	-[a]
BCL2L1/AVEN	-[a]	0.2234	0.0075	-[a]
BCL2L1/RTN4	-[a]	0.2234	0.4258	-[a]
BCL2L1/MOAP1	-[a]	0.2234	0.0448	-[a]
BCL2L1/BCL2L14	-[a]	0.2234	0.0468	-[a]
BCL2L1/BCL2L12	-[a]	0.2234	0.0157	-[a]
BCL2L1/SPNS1	-[a]	0.2234	0.4606	-[a]
BCL2L1/ANTXR1	-[a]	0.2234	0.1310	-[a]
BCL2L1/BMF	-[a]	0.2234	0.0135	-[a]
BCL2L1/BNIPL	-[a]	0.2234	0.0547	-[a]
BCL2L1/vpr	-[a]	0.2234	0.0000	-[a]
BCL2L1/tat	-[a]	0.2234	0.0000	-[a]

Table S4: (continued)

PPI	Druggability score			
	Structural attributes	Drug/chemical attributes	Functional attributes	All attributes
BCL2L1/vpu	- [a]	0.2234	0.0000	- [a]
BCL2L1/env	- [a]	0.2234	0.0000	- [a]
BCL2L1/nef	- [a]	0.2234	0.0000	- [a]
BCL2L1/SP8	- [a]	0.2234	0.0233	- [a]

Druggability score calculated by the SVM-based method using structural, drug/chemical, functional, or all attributes are shown.

[a] '-' means that the druggability score can not be calculated because any pocket overlapped with PPI interface was not detected, thus structural attributes of pocket can not be obtained. In these cases, the druggability score using all attributes is also not calculated.
